# Impact of the Kocher maneuver on anastomotic leak after esophagogastrostomy in combined thoracoscopic-laparoscopic esophagectomy

**DOI:** 10.20407/fmj.2018-011

**Published:** 2019-02-06

**Authors:** Kenichi Nakamura, Koichi Suda, Hokuto Akamatsu, Susumu Shibasaki, Masaya Nakauchi, Kenji Kikuchi, Shinichi Kadoya, Kazuki Inaba, Ichiro Uyama

**Affiliations:** 1 Division of Upper GI, Department of Surgery, Fujita Health University, School of Medicine, Toyoake, Aichi, Japan; 2 Department of Radiology, Fujita Health University, School of Medicine, Toyoake, Aichi, Japan

**Keywords:** Anastomotic leak, Anastomosis, Surgical, Esophagectomy, Esophageal neoplasm, Indocyanine green

## Abstract

**Objectives:**

Anastomotic leak is a common complication after esophagectomy for esophageal cancer. This study evaluated the impact of the Kocher maneuver on the incidence of anastomotic leak following esophagogastrostomy using a 3-cm-wide gastric conduit.

**Methods:**

This single-institution, retrospective, cohort study included 43 patients who underwent thoraco-laparoscopic esophagectomy. The Kocher maneuver was not performed in the first half of the study period between April 2014 and May 2015 (first half group, n=14), but was performed in the second half between May 2015 and January 2017 (second half group, n=29). Primary endpoint was the incidence of anastomotic leak. Metrological values of the gastric conduit were postoperatively assessed on computed tomography. Blood perfusion of the gastric conduit was prospectively examined using the indocyanine green fluorescence method.

**Results:**

The incidence of anastomotic leak was 14%; the incidence was significantly lower in the second half group than in the first half group (3.4% vs. 35.7%, p=0.01). The Kocher maneuver was the only significant independent risk factor associated with anastomotic leak (OR 0.064, 95% CI 0.007–0.625, p=0.018). The postoperative length of the entire gastric conduit was significantly shorter in the second half group than in the first half group. A more anal location of the 3-cm-wide gastric conduit was associated with better blood perfusion.

**Conclusions:**

The Kocher maneuver may enable shortening of the gastric conduit, leading to better blood perfusion of the tip of the gastric conduit, and a significant reduction in the occurrence of anastomotic leak.

## Introduction

One of the most common complications after esophagectomy is anastomotic leak, with a reported incidence of approximately 15%.^1–4^ Since 2006, combined prone-thoracoscopic and supine-laparoscopic esophagectomy has been performed at our institution. Our previous study reported that the incidence of anastomotic leak after esophagectomy in our institution is as high as 22%.^5^ This complication may cause extension of hospitalization and increase mortality.

The right gastroepiploic artery is responsible for supplying blood to the 3-cm-wide greater curvature gastric conduit. A reduction in the length of the conduit may improve blood perfusion at the anastomotic site, thus reducing the likelihood of anastomotic leak. The Kocher maneuver is the dissection of the lateral peritoneal attachments of the duodenum to enable inspection of the duodenum, pancreas, and other retroperitoneal structures over to the great vessels.^6^ This surgical maneuver may be used to attenuate the required length of the gastric conduit. Thus, we started to use the Kocher maneuver in esophagectomy from 2015. The aim of the present study was to evaluate the impact of the Kocher maneuver on the incidence of anastomotic leak after esophagogastrostomy using a 3-cm-wide gastric conduit.

## Materials and Methods

### Patients

The present study was conducted at a single institution. We performed a retrospective review of our prospectively maintained database that included data from 45 consecutive patients with resectable esophageal squamous cell carcinoma who were referred to our department between 2014 and 2016. Forty-three patients who underwent thoraco-laparoscopic esophagectomy were identified and included in the present study; the remaining two patients who underwent cervical esophagectomy were excluded. The Kocher maneuver was not performed in the first half of the study period between April 2014 and May 2015 (first half group, n=14), but was performed in the second half between May 2015 and January 2017 (second half group, n=29). The two groups were compared regarding patient background characteristics, surgical outcomes, and short-term postoperative outcomes (including postoperative complications).

In the thoracic phase, thoracoscopic transthoracic esophagectomy in the prone position was performed. Patients who agreed to the use of the da Vinci Surgical System without insurance underwent robotic esophagectomy (n=13), while the remaining patients underwent the same operation without robotic assistance but with health insurance coverage (n=30). R0 resection with total mediastinal lymphadenectomy was achieved in all patients. The assessed surgical outcomes included total operative time, laparoscopy time, estimated blood loss, postoperative complications, length of postoperative hospital stay, and clinicopathological characteristics. Total operative time was defined as the time from the start of the thoracic incision to the completion of abdominal and cervical wound closures. Blood loss was estimated by weighing the suctioned blood and blood-soaked gauze. Short-term postoperative complications were defined as clinically relevant complications occurring within 30 days postoperatively that required transfusion, central venous nutrition, or medications other than antiemetics, analgesics, antipyretics, or diuretics, corresponding to a Clavien-Dindo classification grade of II or more.^7,8^ Postoperative complications were classified in accordance with the Japan Clinical Oncology Group Postoperative Complication Criteria based on the Clavien-Dindo Classification version 2.0.^9^

The primary endpoint was the incidence of anastomotic leak after esophagogastrostomy, including leak at esophagogastrostomy and leak at the stump of the gastric conduit.

All operations were performed or guided by a single surgeon (I.U.) who had performed more than 100 totally thoracoscopic esophagectomy procedures. The thoracic phase was performed by two surgeons (I.U. and K.S), while the abdominal phase was performed by nine surgeons who had each performed more than 50 laparoscopic gastrectomy procedures. All surgeons were certified by the Japan Society for Endoscopic Surgery via the Endoscopic Surgical Skill Qualification System.

Details of physical function assessment, preoperative cancer staging, laryngopharyngeal function assessment, preoperative treatment, and perioperative management have been reported previously.^5^ All patients received broad-spectrum antibiotics for 48 h postoperatively during hospitalization. Enteral feeding was performed via surgically-placed jejunostomy from postoperative day 1. Patients were discharged when their condition was optimal. The discharged patients visited our outpatient clinic at least after 1 month, and then every 3 months until 5 years postoperatively. Outpatient visits involved physical examinations and regular laboratory assessments, including evaluation of squamous cell carcinoma-related antigen, p53, and carcinoembryonic antigen. To detect local recurrence and systemic metastasis, the patients underwent neck, chest, and abdominopelvic computed tomography (CT) at least within 1 month postoperatively, and then every 6 months. Upper gastrointestinal endoscopy was performed annually to screen for local recurrence and metachronous multicentric or multiple cancers. Patients were involved in the decision-making process, and informed consent for surgery was obtained from all patients. This study was approved by the Institutional Review Board of Fujita Health University.

### Surgical procedures

Details of the operative procedures have been reported previously.^5^ Thoracoscopic esophagectomy was performed in the prone position using six right thoracic ports. Abdominal lymph node dissection and gastric mobilization were performed laparoscopically using five abdominal ports. The right gastric artery was ligated at the second or third branch. Mini-laparotomy was performed at the upper abdominal midline in the supine position. A 3-cm-wide greater curvature gastric conduit was extracorporeally created using linear staplers. A slanting incision was made in the left side of the neck for dissecting the cervical lymph nodes and for esophagogastrostomy. The gastric conduit was pulled up through the retrosternal route and extracted through the neck incision for anastomosis. Esophagogastrostomy was performed via end-to-end hand-sewn anastomosis, end-to-end triangular anastomosis using a linear stapler,^10,11^ or end-to-side anastomosis using a circular stapler. In end-to-side anastomosis, a circular stapler was inserted through a gastrostomy at the tip of the gastric conduit, the anvil of the stapler was inserted into the remnant esophagus, and anastomosis was performed at the greater curvature side of the stomach. After firing, the tip of the gastric conduit was closed from the greater curvature to the lesser curvature using a linear stapler at 3 cm cranially to the esophagogastrostomy. The staple line was inverted with sutures to prevent its adhesion to the surrounding tissue. The type of anastomosis was selected in accordance with the surgeon’s preference. Finally, jejunostomy was performed 20 cm distal from the Treitz ligament via a laparoscopy-assisted procedure.

### Kocher maneuver

**Figure 1:**
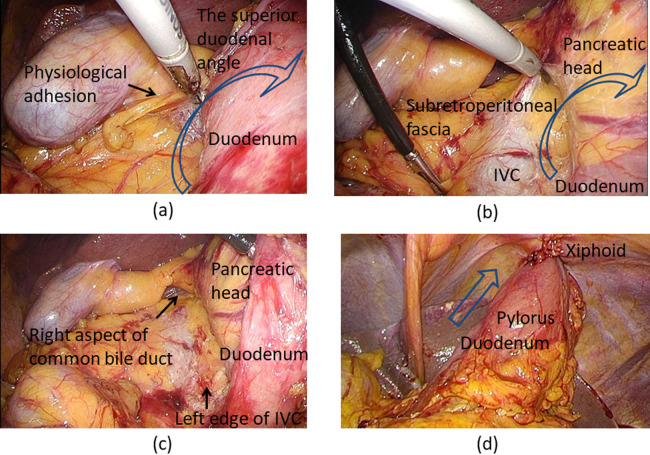
Kocher maneuver. (a) The assistant surgeon pulls the duodenum up and left from the patient’s left side (arrow), and the operating surgeon removes the physiological adhesions on the cranial aspect from the patient’s right side. (b) The duodenum in combination with the pancreatic head is extensively mobilized (arrow) on the subretroperitoneal fascia. (c) The landmarks of sufficient mobilization are exposure of the left edge of the inferior vena cava and the right aspect of the common bile duct. (d) The Kocher maneuver results in sufficient elevation of the 3-cm-wide gastric conduit toward the xiphoid, which is the entrance of the retrosternal route (arrow). IVC, inferior vena cava

The Kocher maneuver is the procedure to mobilize the duodenum from the retroperitoneum. After mobilizing the hepatic flexure of the colon from the ventral aspect of the duodenum, the superior duodenal angle was fully mobilized by removing physiological adhesions on its cranial aspect (Figure 1a). The duodenum and the pancreatic head were extensively mobilized on the subretroperitoneal fascia (Figure 1b). The landmarks of sufficient mobilization were exposure of the left edge of the inferior vena cava (Figure 1b) and the right aspect of the common bile duct (Figure 1b, 1c). The accessary right colic vein was ligated and cut in all cases.

### Metrological values of the gastric conduit after esophagogastrostomy

**Figure 2:**
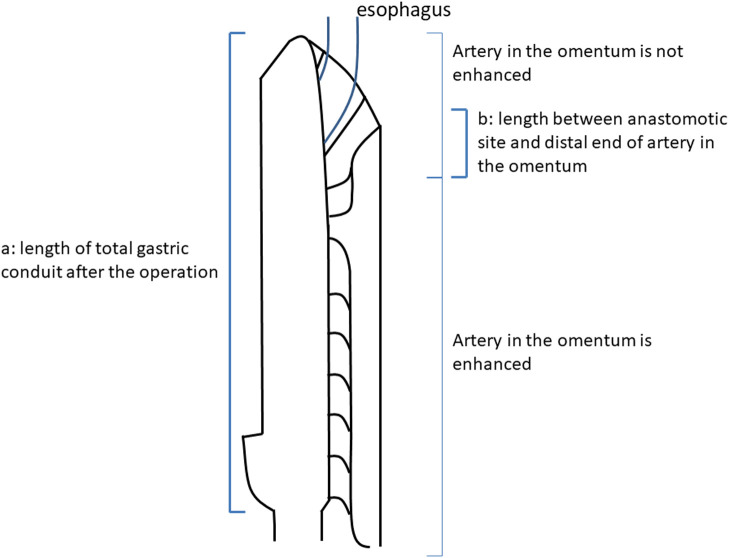
Measured values of the 3-cm-wide gastric conduit on postoperative computed tomography.

**Figure 3:**
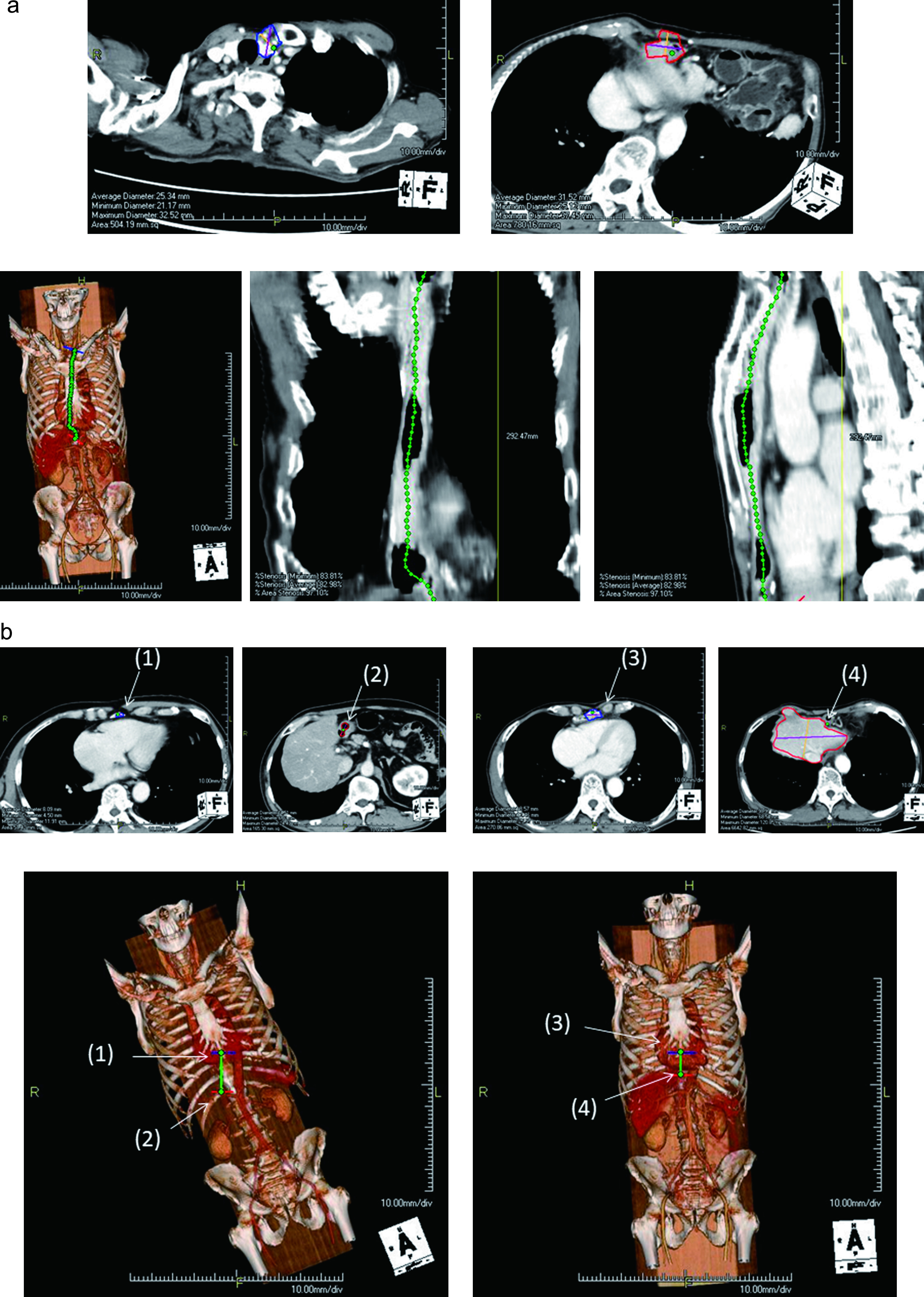
The Aquarius NET Server (TeraRecon, San Mateo, CA, USA). (a) Method for measuring the length of the gastric conduit after the operation. The length is measured by plotting the center of the gastric conduit between objects in the axial section for every 5-mm slice. (b) Postoperative shortening of the distance between the xiphoid and the pylorus. (1) Preoperative location of the xiphoid; (2) preoperative location of the pylorus; (3) postoperative location of the xiphoid [same as (1)]; (4) postoperative location of the pylorus. Postoperative shortening of the distance between the xiphoid and pylorus is measured by subtracting the distance between (3) and (4) from the distance between (1) and (2).

Enhanced CT was performed within 1 month postoperatively in all 43 patients. The length of the entire gastric conduit after the operation (Figure 2a) and the length of the gastric conduit between the anastomotic site and the distal end of the artery in the omentum (distal end of the enhanced artery from the right gastroepiploic artery) (Figure 2b) were assessed using the Aquarius NET Server (TeraRecon, San Mateo, CA, USA) (Figure 3).^12–15^ The values were measured by plotting the center of the gastric conduit between objects in the axial section for every 5-mm slice (Figure 3a, upper images). Those plotted points were then converted to a line in the 3D image, enabling the measurement of even a curved distance between the target points with little error (Figure 3a, lower images). In addition, the postoperative shortening of the distance between the xiphoid and pylorus was assessed by comparing the pre- and postoperative distances between the xiphoid, which was the entrance of the retrosternal route, and the pylorus (Figure 3b).

### Blood perfusion in the gastric conduit

**Figure 4:**
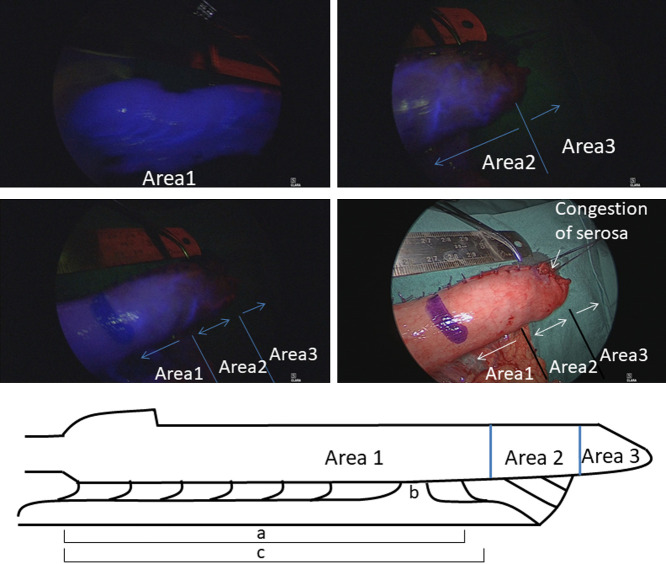
Blood perfusion of the 3-cm-wide gastric conduit as determined using the indocyanine green fluorescence method. Area 1, good perfusion; area 2, congestion; area 3, no perfusion Area 1 is promptly perfused, and the entire gastric wall is dyed. Area 2 is dyed more slowly than area 1, and only the blood vessels in the gastric wall are dyed. Area 2 is almost the same as a macroscopically congested site. In area 3, no dyeing is seen. (a) Length between the pylorus and the first branch of the left gastroepiploic artery. (b) Watershed. (c) Length of the gastric conduit after anastomosis.

The blood perfusion of the 3-cm-wide gastric conduit was prospectively examined using the indocyanine green (ICG) fluorescence method in a series of six patients who underwent thoraco-laparoscopic esophagectomy for esophageal cancer between October and November 2017. In brief, after the creation of a 3-cm-wide greater curvature gastric conduit extracorporeally (as described above), the anesthesiologist injected 0.5 mg/kg of ICG dye through peripheral vessels. NIR/ICG fluorescence imaging (KarlStorz, Tuttlingen, Germany) was used to visualize fluorescence. The video camera was placed 5 cm above the gastric conduit, and the fluorescence image was recorded from before the injection of ICG to 120 s after the dyeing of the root of the right gastroepiploic artery. Perfusion of the gastric conduit was evaluated after the fluorescence of the gastric conduit had reached a steady state. The length between the pylorus and the first branch of the left gastroepiploic artery (Figure 4a), the connection of the watershed (area between the right and left gastroepiploic arteries) (Figure 4b), and the length of the gastric conduit after anastomosis (Figure 4c) were determined. The extent of blood perfusion was qualitatively divided into the following three areas in accordance with the findings on visual inspection: area 1, good perfusion; area 2, congestion; area 3, no perfusion.

### Statistical analysis

Between-group comparisons were performed using a χ^2^ or Mann-Whitney *U* test. A univariate χ^2^ test and a multivariate logistic regression analysis with backward stepwise elimination were used to determine the factors associated with the occurrence of postoperative complications. Considering the relatively small sample size, all variables with a significance level of p<0.05 in the univariate analysis for surgical outcomes were included as independent variables in the multivariate analysis. Data are expressed as median (range) or odds ratio (OR) with 95% confidence interval (CI), unless otherwise stated. All analyses were performed using IBM SPSS Statistics 21 (IBM Corporation, Armonk, NY, USA). A two-tailed p value of <0.05 was considered statistically significant.

## Results

### Patient background characteristics

Patient background characteristics are summarized in Table 1. There were no significant differences in the demographic and oncological backgrounds between the study groups. The HbA1c level was less than 6.5% in most patients, and only three patients (7%) had an HbA1c level greater than 6.5%.

### Operative procedure, short-term surgical outcomes, and postoperative course

There was a significant difference between groups in the anastomotic procedure used (Table 2); all esophagogastrostomy procedures in the second half group were performed using a circular stapler, whereas nine of 14 patients in the first half group received a circular-stapled anastomosis. Compared with the first half group, the second half group had a significantly longer laparoscopy time and a significantly shorter duration of postoperative hospitalization. There were no differences between the two groups in the robotic thoracoscopic surgery rate, robotic laparoscopic surgery rate, total operative time, and estimated blood loss.

### Metrological values of the gastric conduit

The metrological values on CT of the gastric conduit created with or without the Kocher maneuver are summarized in Table 2. Compared with the first half group, the second half group had a significantly shorter length of the entire gastric conduit (p=0.005), and significantly shorter length of the gastric conduit between the anastomotic site and the distal end of the artery in the omentum (p=0.016). The postoperative shortening of the distance between the xiphoid and pylorus was significantly greater in the second half group than in the first half group (p=0.032).

### Short-term postoperative complications

The short-term postoperative complications are summarized in Table 3. The total incidence of anastomotic leak was 14%. The incidence of anastomotic leak was significantly lower in the second half group than in the first half group (p=0.01). In the first half group, anastomotic leak occurred at the anastomotic site in two patients who underwent hand-sewn (end-to-end) anastomosis and one who underwent triangular (end-to-end) anastomosis. Moreover, of the two patients in the first half group who underwent circular stapler (end-to-side) anastomosis, one developed a leak at the stump of the gastric conduit, while the other had a leak at an unknown location. In the second half group, one patient who underwent circular stapler (end-to-side) anastomosis had a leak at the stump of the gastric conduit. Overall, five patients had an anastomotic leak at the tip of the gastric conduit, while one had a leak at an unknown location. The rate of anastomosis-related complications was significantly lower in the second half group than in the first half group. However, the incidences of other complications did not differ between the groups.

### Factors associated with anastomotic leak

To determine the factors associated with anastomotic leak, univariate analysis was performed using the following variables: age, sex, body mass index, tumor size, tumor location, preoperative albumin level, clinical Japanese Classification of Gastric Carcinoma stage, pathologic Japanese Classification of Gastric Carcinoma stage, American Society of Anesthesiologists Physical Status, history of laparotomy, comorbidity, diabetes mellitus, hypertension, chronic renal disorder, preoperatively high creatinine level (0.85 mg/dl), corticosteroid use, current smoking, preoperative therapy, neoadjuvant chemotherapy use, preoperative chemoradiation therapy, robot use for the abdominal surgery, robot use for the chest surgery, total operative time, laparoscopy time, estimated blood loss, transfusion, preservation of the thoracic duct, Kocher maneuver, length of the gastric conduit between the anastomotic site and the distal end of the artery, length of the entire gastric conduit, combined resection, and anastomotic procedure (Table 4). The factors significantly associated with anastomotic leak were the Kocher maneuver (p=0.01), length of the entire gastric conduit (p<0.001), and anastomotic procedure (p=0.014). There was a significant association between the Kocher maneuver and the length of the entire gastric conduit (p=0.005). Subsequently, multivariate analysis was performed using the variables with a significance level of p<0.05 in the univariate analysis (Kocher maneuver and anastomotic procedure). In the multivariate analysis, the Kocher maneuver was the only significant independent risk factor associated with anastomotic leak (OR 0.064, 95% CI 0.007–0.625, p=0.018) (Table 4). However, of the patients who underwent circular stapled anastomosis, there was no significant difference between the first and the second half groups in the incidence of anastomotic leak (p=0.134).

### Blood perfusion of the gastric conduit

The measured values of each 3-cm-wide gastric conduit in the six assessed cases are shown in Table 5. The Kocher maneuver and anastomosis using a circular stapler were performed in all cases. Connection of the watershed was noted in four cases. It took 50.5 (range, 40–90) s for the gastric conduit to reach a steady state. As expected, a more anal site was associated with better blood perfusion (Table 5, Figure 4). Area 1 was promptly perfused and the entire gastric wall was dyed; this area extended up to a point slightly proximal to the first branch of the left gastroepiploic artery from the pylorus, irrespective of the watershed connection. Area 2 was dyed more slowly than area 1, and only blood vessels in the gastric wall were dyed; this area was almost the same as the macroscopically congested site. In area 3, no dyeing was seen. Both the esophagogastrostomy and the stump of the gastric conduit after anastomosis were located in area 1 in five cases (83%), and in area 2 in one case (17%). No anastomotic leak occurred in any of the six cases.

## Discussion

The present study clearly demonstrated a positive association between the performance of the Kocher maneuver during combined thoracoscopic-laparoscopic esophagectomy and a reduction in anastomotic leak after esophagogastrostomy.

In the present study, the Kocher maneuver was the only significant independent risk factor associated with anastomotic leak. To the best of our knowledge, this is the first report to suggest that the Kocher maneuver may be an effective method to prevent anastomotic leak. Diabetes mellitus, preoperative hypertension, high creatinine level (>0.85 mg/dl), corticosteroid use, and current smoking were not risk factors for anastomotic leak, and these results were inconsistent with the findings of previous studies.^16,17^ This may be partly because these factors were preoperatively controlled by physicians for most of the patients in the present study.

Retrospective examination of postoperative CT images revealed that the Kocher maneuver reduced the length of the gastric conduit by approximately 2 cm. Moreover, the prospective ICG fluorescence study showed that a more anal site of the gastric conduit was associated with better blood perfusion. This is at least partly because the blood perfusion of the 3-cm-wide gastric conduit is mostly associated with the right gastroepiploic artery. The ICG fluorescence study showed that the esophagogastrostomy and the stump of the gastric conduit after anastomosis were located in area 1 in five of the six assessed cases. However, if the Kocher maneuver had not been performed, the stump of the gastric conduit in four of the six cases would have been in area 2, although the area of the esophagogastrostomy would not have changed. In the patients who underwent circular stapled anastomosis, the distance between the tip of the gastric conduit and the anastomotic site was actually set as 3 cm. Although we did not use the ICG fluorescence method to examine the blood perfusion at the anastomotic sites of hand-sewn and triangular anastomoses (which were created in the end-to-end manner), those sites were expected to be located within 3 cm from the tip of the conduit. Therefore, the Kocher maneuver may have improved the perfusion of the tip of the gastric conduit, resulting in decreased occurrence of anastomotic leak. Furthermore, area 2 was located more cranial to the first branch of the left gastroepiploic artery, and area 2 was macroscopically congested. Collectively, these findings suggest that both the esophagogastrostomy and the stump of the gastric conduit should ideally be created in area 1, and both the macroscopically ischemic part and the congested part of the gastric conduit should be removed.

To assess the blood perfusion of the gastric conduit using ICG fluorescence with visual inspection, the dose of ICG should be about 10 times greater than that used in previous reports.^18–20^ In fact, some of the present cases required optimization of the dose before starting this study. We do not consider this dose to be excessive, as the same dose has long been clinically used for liver function tests.^21^

The present study has several limitations. First, the study was conducted in a single institution with a non-randomized design. Therefore, the data might be biased, and the overall results should be interpreted cautiously. Second, multivariate analyses may not fully remove the impact of the learning effect and the between-group difference in anastomotic procedures. Notably, the Kocher maneuver was only performed in the second half group. Additionally, in patients with circular stapled anastomosis, there was no difference in the incidence of anastomotic leak between the first and the second half groups, although this may be at least partly due to the small sample sizes. Third, although the Kocher maneuver resulted in better gastric conduit elevation, the main trunk of the right gastric artery prevented mobility of the gastric conduit in some cases, even when the Kocher maneuver was used.

In conclusion, the Kocher maneuver may be an effective additional procedure to reduce anastomotic leak after esophagogastrostomy. A prospective, larger trial is warranted to confirm the present findings.

## Figures and Tables

**Table1:** Patient background characteristics

Number of cases	All 43	The first half group 14	The second half group 29	p-value
Age	69 (51–79)	69 (51–73)	69 (54–79)	0.533
Sex (M:F)	37:6	12:2	25:4	0.649
Body mass index (kg/m^2^)	23.9	24.3	23.9	0.959
Preoperative albumin (g/dl)	4.2 (3.0–4.8)	4.2 (3.1–4.8)	4.1 (3.0–4.7)	0.211
ASA-PS (1:2:3)	15:25:3	6:7:1	9:18:2	0.769
Comorbidity, n (%)	32 (74.4)	10 (71.4)	22 (75.9)	0.515
Diabetes mellitus, n (%)	7 (16.3)	1 (7.1)	6 (20.7)	0.255
Preoperative HbA1c	5.8 (4.7–6.6)	5.8 (5.1–6.6)	5.7 (4.7–7.6)	0.28
Steroid use, n (%)	3 (7.0)	1 (7.1)	2 (6.9)	0.704
Chronic renal disorder, n (%)	5 (11.6)	3 (21.4)	2 (6.9)	0.186
Preoperative therapy, n (%)	21 (48.8)	7 (50)	14 (48.3)	0.916
Neoadjuvant chemotherapy use, n (%)	15 (34.9)	5 (35.7)	10 (34.4)	0.598
Preoperative chemo-radiation therapy use, n (%)	6 (14.0)	2 (14.3)	4 (13.8)	0.649
Pathological JCGC stage (0:I:II:III:IV)	5:4:16:16:2	2:2:3:5:2	3:2:13:11:0	0.255

First half group, patients who underwent esophagogastrostomy without the Kocher maneuver in the period between April 2014 and May 2015 (n=14); second half group, patients who underwent esophagogastrostomy with the Kocher maneuver in the period between May 2015 and January 2017 (n=29); ASA-PS, American Society of Anesthesiologists Physical Status; JCGC, Japanese Classification of Gastric Carcinoma

**Table2:** Operative procedure, short-term surgical outcomes, and postoperative course

	All	The first half group	The second half group	p-value
Operative procedure				
Robotic thoracoscopic surgery, n (%)	13 (30.2)	7 (50)	6 (20.7)	0.056
Robotic laparoscopic surgery, n (%)	2 (4.7)	1 (7.1)	1 (3.5)	0.55
Anastomotic procedure (hand-sewn:triangular anastomosis:circular stapler)	3:2:38	3:2:9	0:0:29	0.002
Short-term surgical outcome				
Total operative time (min)	710 (456–1110)	689 (561–803)	718 (456–1110)	0.17
Laparoscopy time (min)	181 (67–338)	128 (103–198)	182 (67–338)	0.013
Estimated blood loss (g)	136 (35–925)	120 (35–340)	161 (45–925)	0.087

Length of the entire gastric conduit after operation (from its stump to the pylorus) (mm)	293 (215–358)	309 (272–358)	282 (215–356)	0.005
Length between the anastomotic site and distal end of the artery in the omentum (mm)	0 (0–134)	43 (0–134)	0 (0–131)	0.016
Shortening of the distance between the xiphoid and pylorus after the operation (mm)	60 (8–108)	45 (8–108)	64 (11–105)	0.032

Postoperative courses				
Length of postoperative hospital stay (days)	34 (14–194)	46 (20–194)	31 (14–68)	0.036

First half group, patients who underwent esophagogastrostomy without the Kocher maneuver in the period between April 2014 and May 2015 (n=14); second half group, patients who underwent esophagogastrostomy with the Kocher maneuver in the period between May 2015 and January 2017 (n=29).

**Table3:** Postoperative complications of thoraco-laparoscopic esophagectomy

Postoperative complication	All	The first half group	The second half group	p-value
Within 30 days following surgery C-D grade ≥II, n (%)				
Morbidity, n	46	22	24	0.437
Anastomosis related, n	7	5	2	0.028
Anastomotic leak, n (%)	6 (14.0)	5 (35.7)	1 (3.4)	0.01
Anastomotic stenosis, n (%)	1 (2.3)	0	1 (3.4)	0.674
Anastomotic bleeding, n (%)	0	0	0	

First half group, patients who underwent esophagogastrostomy without the Kocher maneuver in the period between April 2014 and May 2015 (n=14); second half group, patients who underwent esophagogastrostomy with the Kocher maneuver in the period between May 2015 and January 2017 (n=29); C-D, Clavien-Dindo.

**Table4:** Factors associated with anastomotic leak

	Univariate analysis	Multivariate analysis	OR (95%CI)
	p-value	p-value	
Age	0.878		
Sex	0.619		
Body mass index	0.745		
Tumor size	0.104		
Location	0.145		
Preoperative albumin	0.644		
Clinical JCGC stage	0.106		
Pathologic JCGC stage	0.083		
ASA-PS	0.782		
History of laparotomy	0.63		
Comorbidity	0.488		
Diabetes mellitus	0.319		
Hypertension	0.547		
Chronic renal disorder	0.453		
Preoperative high creatinine level (>0.85 mg/dl)	0.221		
Steroid use	0.63		
Current smoking	0.547		
Preoperative therapy	0.645		
Neoadjuvant chemotherapy use	0.304		
Preoperative chemo-radiation therapy use	0.19		
Robot use for the abdomen	0.262		
Robot use for the chest	0.058		
Total operative time	0.327		
Laparoscopy time	0.262		
Estimated blood loss	0.986		
Transfusion	0.63		
Preservation of the thoracic duct	0.571		
Kocher maneuver	0.01	0.018	0.064 (0.007–0.625)
Length of the entire gastric conduit	<0.001		
Length of the gastric conduit between the anastomotic site and distal end of the artery	0.16		
Combined resection	0.619		
Anastomotic procedure	0.014		

ASA-PS, American Society of Anesthesiologists Physical Status; JCGC, Japanese Classification of Gastric Carcinoma

**Table5:** Indocyanine green fluorescence in the 3-cm-wide greater curvature gastric conduit

Patient No.	Area 1 (cm)	Area 2 (cm)	Area 3 (cm)	Pylorus-first branch of the left gastroepiploic artery (cm)	Connection of the watershed	Pylorus-esophagogastrostomy (cm)	Length of the gastric conduit after anastomosis (cm)	Esophagogastrostomy (area)	Gastric stump (area)
1	0–25	25–28	28–30	23.5	−	22	25	1	1
2	0–20	20–30	30–32	18	−	21	24	2	2
3	0–28	28–30	30–32	24	+	23	25	1	1
4	0–29	29–35	—	26	+	25	28	1	1
5	0–30	30–36	—	24	+	24.5	27.5	1	1
6	0–25	25–31	31–33	25	−	21.5	24.5	1	1

Area 1, good perfusion; area 2, congestion; area 3, no perfusion.
